# Contribution of the patient–horse relationship to substance use disorder treatment: Patients’ experiences

**DOI:** 10.3402/qhw.v11.31636

**Published:** 2016-06-09

**Authors:** Ann Kern-Godal, Ida H. Brenna, Norunn Kogstad, Espen A. Arnevik, Edle Ravndal

**Affiliations:** 1 Department of Addiction Treatment, Oslo University Hospital, Oslo, Norway; 2 Norwegian Centre for Addiction Research, University of Oslo, Oslo, Norway; 3 Reinsvoll Psychiatric Hospital, Sykehuset Innlandet, Reinsvoll, Norway; 4 Department of Psychology, University of Oslo, Oslo, Norway

**Keywords:** Substance use disorder, addiction, patient perspective, human–horse relationship, therapeutic relationship, attachment, qualitative

## Abstract

**Background:**

A good therapeutic relationship is a strong predictor of successful treatment in addiction and other psychological illness. Recent studies of horse-assisted therapy (HAT) have drawn attention to the importance of the client's relationship to the horse in psychotherapy. Few have reported on the patient's own perspective and none have reported specifically on the human–horse relationship in substance use disorder (SUD) treatment and its implications for health and well-being.

**Aim:**

This article explores SUD patients’ own experience of their relationship with the horse and their perceptions of its contribution to their therapy.

**Methods:**

As part of a large mixed-method study of HAT in SUD treatment, we used semi-structured interviews of eight patients to gather information about their experiences of HAT. From the data obtained, the relationship with the horse was found to be a significant part of participants’ HAT experience. It is therefore the subject of the current phenomenological study, in which thematic analysis was used to investigate how the participants constructed the reality of their relationship with the horse(s) and their perceptions of the consequences of that reality in SUD treatment.

**Results:**

Participants’ own descriptions suggest that the horses were facilitators of a positive self-construct and provided important emotional support during treatment. Analysis found *relationship with the horse*, *emotional effect*, and *mastery* to be important and interrelated themes. The findings were interpreted within an attachment theory context.

**Conclusion:**

The results appear to be consistent with key addiction treatment theories and with findings in HAT theoretical and empirical studies. They add to our understanding of the impact of HAT on SUD treatment. However, further research is needed into both the construct validity of the patient–horse therapeutic relationship and the possible variance within and between different populations.

There is a continuous struggle to find treatment modalities that motivate substance use disorder (SUD) patients to remain in treatment for sufficient time to enable beneficial change in morbidity (Dalsbø et al., 2010). Treatment factors such as time in treatment, treatment ideology, patient staff ratios, and patient–therapist alliance have been found to be among the best predictors of treatment outcome (Brorson, Ajo Arnevik, Rand-Hendriksen, & Duckert, 2013).

While early therapeutic alliance is said to predict retention in treatment (Meier, Donmall, McElduff, Barrowclough, & Heller, 2006), alliance is generally considered to be a “common factor” found in many different treatments (Laska, Gurman, & Wampold, 2014; Messer & Wampold, 2002). Miller and Moyers (2014) examined “specific” and “common factors” in addiction treatment and concluded that specific treatment methods yield small to no difference, whereas relational factors, such as empathy and alliance, can be significant determinants of treatment outcome. They advocated investigation of treatment processes or “mechanisms of action” that might foster relationship and alliance building (Miller & Moyers, 2014, p. 401). Similarly, others have identified attachment and in-therapy experiences as areas of SUD treatment requiring further study (Flores, 2006; Roth & Fonagy, 2013).

Regardless of theoretical basis and terminology, *horse-assisted therapy* (HAT) can be defined as psychotherapy that in some way or another includes horses. Practitioners of HAT such as Shambo, Young and Madera's (2013), Karol (2007), and Hallberg (2008) claim that HAT presents unique opportunities to work within a three-way client–horse–therapist therapeutic relationship. They believe that interaction with horses brings other dimensions to the therapy. Some of these benefits are believed to be an effect of the sheer size and power of the horse and its capacity to carry the rider (Yorke, Adams, & Coady, 2008). Other suggested benefits are believed to be a result of the inherent characteristics of the horse, such as learning from its herd-based, cooperative behaviour to experience new forms of behaviour and feelings (Burgon, 2014).

The horse is also claimed to be useful as a metaphor; non-judgmental and motivational; useful for building self-esteem, confidence, and mastery; and effective for building trust and attachment with both the horse and the therapist (Bachi, 2013; Burgon, 2014; Koren & Træen, 2003; Selby & Smith-Osborne, 2013). It has also been suggested that social interaction with the horse can shed light on human interactions and its meanings, as well as on possibilities for behaviour change (Hamilton 2011; Koren & Træen, 2003).

Recent reviews of HAT literature, while cautiously optimistic, all draw attention to shortcomings, including the mixed diagnoses of client groups, and the need for improved methodology and theoretical analysis (Anestis, Anestis, Zawilinski, Hopkins, & Lilienfeld, 2014; Bachi, 2012; Lee, Dakin, & McLure, 2016; Lentini & Knox, 2015).

Social well-being is an underlying theme in many studies of therapeutic and non-therapeutic (or recreational) activities with horses. In recent years, general horse discourse has addressed bio-social aspects of human–horse interaction and the accrued well-being of humans resulting from that interaction (Birke & Hockenhull, 2015; Davis & Maurstad, 2016). Similarly, in HAT, the human–horse bond, as well as the patient–horse–therapist triad, have been increasingly explored in terms of relational factors and attachment theory (Bachi, 2013; Burgon, 2014; Fry, 2013; Karol, 2007). However, few have specifically investigated the impact of the human–horse relationship in therapy from the patient's own perspective. A rare exception is Yorke et al.'s (2008) study of the equine–human bond and its contribution to recovery from trauma. Their participants described how their pre-existing relationships with horses were therapeutic during trauma recovery, causing significant change in their lives.

We identified six studies reporting on the perspectives of SUD clients working with horses as part of their therapeutic activities. Only two (Adams et al., 2015; Dell et al., 2011) were in peer-reviewed journals. Both involved First Nations youth who misused solvents. Conducted within a bio–psycho–social–spiritual framework, these programmes were found to improve the social well-being of the youth and relationships within the community. Neither study discussed the client–horse relationship in detail.

## Study context

This study is part of an on-going, mixed-methods, multi-aspect investigation of the impact of HAT provided as an integral part of SUD treatment at an addiction treatment facility in Norway (Kern-Godal, 2013). A quantitative study in 2012 found that HAT participants remained in treatment longer (*p*<0.001) and were significantly more likely to complete treatment (OR 8.4, 95%, CI 2.7–26.4, *p*<0.001) than those not participating in HAT (Kern-Godal, Arnevik, Walderhaug, & Ravndal, 2015). These findings, together with an unreported pilot study, prompted a qualitative investigation of the patients’ own perspectives of HAT as part of SUD treatment (Brenna, 2013). The wide-ranging qualitative study provided good descriptive data on a broad range of HAT-related issues, including rich data on participants’ relationship with the horses. At about the same time we became aware of Bachi's (2013) application of attachment theory to equine-facilitated psychotherapy. This furthered our interest in exploring the significance of the patient–horse relationship in SUD treatment.

The addiction facility where the present study took place offered different levels of treatment and assessment to young adults aged between 16 and 26 years (but patients up to 35 years of age might be accepted) with problems related to substance use. The programme is person-centred, comprising individual and group therapy based on a bio–psycho–social model with emphasis on mentalization-based theory and practice (Skårderud & Sommerfeldt, 2013). Psychological treatment was provided according to the individual's specific problems and treatment goals. All treatment units (diagnostic, day, and in-patient) were situated on the grounds of what was once a traditional nineteenth century psychiatric hospital. Much of the almost rural-like setting had been retained, including the stables, which were adjacent to the residential quarters and from which the horses were visible.

HAT was a carefully planned part of patients’ overall SUD treatment plan. It was provided as therapy and, while it might have been enjoyable, it was neither recreational riding nor instructional. It was based on a body-orientated psychotherapy approach (Young, 2006) within a salutogenic framework (Eriksson & Lindström, 2007; Lindström & Eriksson, 2005) and was provided by experienced psychotherapists, who were also qualified riding instructors and skilled horse handlers. The activities were developed over some years (Lysell, 2011). They were drawn from a number of sources, including traditional Norwegian riding therapy, that is, exercise on and around the horse aiming at physical and cognitive improvement (Trætteberg, 2006). Other sources included trauma therapy, based on Shambo et al.'s (2013) “six keys to relationship,” which teaches core skills for navigating and enhancing relationships, while strengthening autonomy and awareness of self, as well as the Equine Assisted Growth and Learning Association's (EAGALA, 2006) programme of experiential growth and learning groundwork with the horse(s). Participants worked directly with the horses (in grooming, feeding, riding, driving) and might undertake other stable activities (mucking out, moving hay, tack cleaning) in close cooperation with the stable staff.

Quantitative data can provide an indication that a particular therapeutic process appears to be beneficial, but participants, such as the therapists and the patients themselves, can add important insights into why this may or may not be so (Malterud, 2001). There is growing emphasis on investigating patients’ own perspectives to enable better understanding of their needs and to improve provider responsiveness. For example, Laudet (2007) showed different meanings of recovery with implications for clinical and assessment practice, and Neale (1998) demonstrated the importance of obtaining SUD patients’ perspectives on use (or underuse) of SUD services. Similarly, Skatvedt and Schou (2010) showed how patients can contribute new meanings about the importance of everyday communication and interaction in a therapeutic community. Misunderstanding, stigma, and prejudice in both the community and treatment settings (Thornicroft et al., 2016) can lead to inappropriate and underused services, as well as to undesirable premature termination of treatment. The design and conduct of the current study was governed by our need for a better understanding of how the patients themselves perceived the horses’ contribution to their SUD treatment.

## Aim

This qualitative study aimed to explore SUD patients’ own experience of their relationship with the horse(s) and its contribution to their treatment. In doing so we aimed to give voice to the participants in our endeavour to increase knowledge about the patient–horse relationship and our understanding of HAT's impact on SUD treatment processes.

## Material and methods

### Design

To maximize individual participant input and to increase comprehension of the issues from the patients’ perspectives, our interview guide and detailed semi-structured questionnaire were constructed using a framework within which respondents could express their experience and understandings in their own terms (Carlson, 2006; Patton, 2015). The interview focus was broad, namely HAT in the context of SUD. However, there was a specific question about the relationship with the horse(s) and ample additional opportunities during the interview for participants to spontaneously elaborate on this, as well as on the meanings they attributed to that relationship.

### Recruitment

Purposeful sampling (to maximize variations in gender, treatment unit, and number of HAT sessions) was used (Patton, 2015) with the primary criterion of at least 1 h of HAT experience. Snowball sampling was used insofar as some participants talked with others, helping to recruit them into the study. No patient refused the invitation but some HAT participants were not invited because their therapists advised that the interview might be detrimental at that point in their recovery process. The original plan was to interview two patients from each of the three treatment units. Unfortunately, at the time only one day patient participated in HAT. An additional two participants were included to test saturation, but because little additional information was obtained no further participants were sought.

### Participants

The eight participants were recruited from amongst 16 young adults at a SUD treatment facility who participated in HAT between late 2012 and early 2013. Four participants were male and four were female; all were aged between 20 and 30 years (mean 25 years). Four entered treatment with comorbid conditions (two with ADHD, one with depression, and one with personality disorder). At the time of the interviews, the participants were at different stages in their treatment programme. Two were residents at the assessment/intermediate unit, five were residents at the in-patient unit, and one attended the day-treatment unit. None were under mandatory court or legislative sanction. Three were experienced riders. The others had had no experience with horses prior to HAT.

### The researchers

Researchers’ own backgrounds, situations, and interests influence study choices and the research methods used (Malterud, 2001). The authors of this paper include a mix of relevant academic (sociology, psychology, and medicine) and practice (addiction treatment, health management, and medical) backgrounds. The first three authors have owned and worked with horses in various capacities for many years. Their co-authors are constantly in search of innovative means to improve SUD treatment and retention. The first author worked as an assistant in the HAT programme from 2007 to 2010. She is also familiar with the broad spectrum of international horse therapy literature and developments. In 2012 she pilot-tested a semi-structured questionnaire-based interview in English with four patients. She concluded that a Norwegian interviewer, closer in age and background and less well known to the participants, would add validity. The second author was therefore recruited.

### The theoretical framework

Wanting to know how patients construct and interpret their experience of the patient–horse relationship as part of SUD therapy, we adopted a phenomenological approach to investigate participants’ own understanding of their relationship to the horses and their shared assumptions about its meaning and reality within SUD treatment (Burr, 1995; Cromby & Nightingale, 1999; Patton, 2015). In doing so, we aimed to give a “voice to the ‘subjects’ we are trying to learn more about” (Carlson, 2006, p. 201). We adopted a position of ontological reality and subjective epistemology, namely an understanding of reality of the patient–horse relationship, not as a unique “truth,” but as derived from shared meanings in the participants’ accounts (Patton, 2015). Attachment theory (Bowlby, 1988; Wallin, 2007) provided the frame for our examination of the SUD (Flores, 2006) and the HAT (Bachi, 2013) related findings and their implications.

### Data collection and analysis

Data on patients’ perceptions of both the horse specific and other aspects of HAT were collected over a 10-week period from late November 2012 to late January 2013. The semi-structured interviews were conducted by the second author within the stable/department environment and recorded on a digital audio recorder. They were transcribed, coded (using HyperRESEARCH, Researchware, Inc., Randolph, MA, USA), and analysed in Norwegian using thematic analysis based on Braun and Clarke's (2006) six suggested steps. The outcome from this analysis was presented to three of the participants who were still in treatment and to the HAT staff. This resulted in some factual elaboration but no change in content (Brenna, 2013).

**Figure 1:**
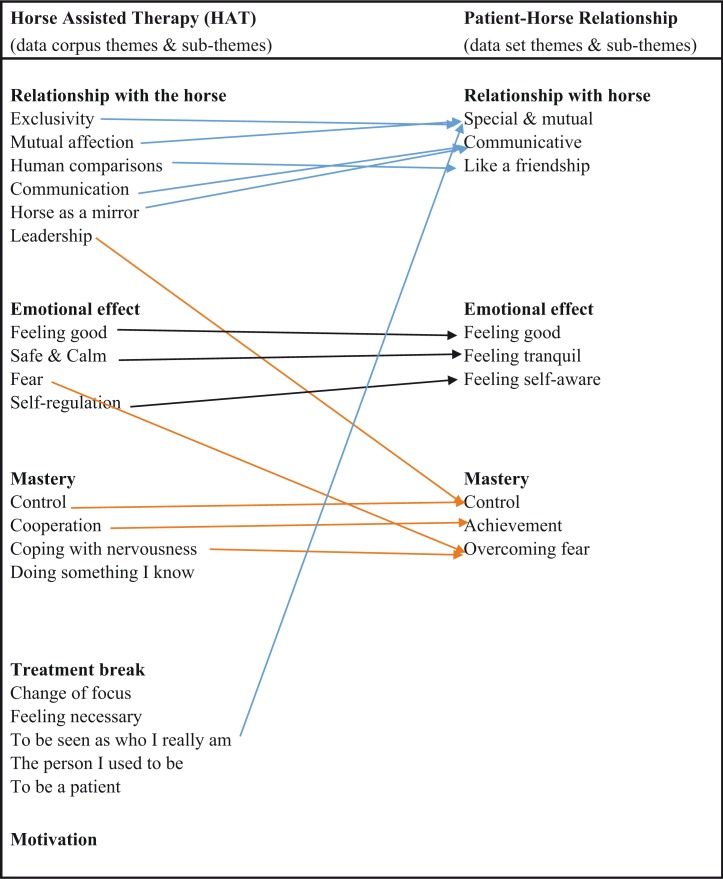
Identification of patient–horse relationship thematic patterns.

The initial analysis referred to above showed the participants’ relationships with horses to be a major theme. The full transcripts for each participant were then reviewed by the first three authors, working independently. They read and re-read all transcripts to identify their “dataset” (Braun & Clarke, 2006, p. 79), namely the material specifically relevant to the relationship with the horses and meanings attributed to that relationship. Thematic analysis was used to “identify patterns across the dataset in relation to the research question” (Braun & Clarke, 2014, p. 2). They then compared the patterns they had each identified from the coded material. Agreement was reached on three themes and nine sub-themes (Fig. 1).

In addition, the first author reviewed video recordings of three of the participants (available from another study), plus three available HAT patient notebooks (patients’ own records of each HAT session). These were found to be consistent with the described/observed relationship with the horses and provided little additional insight. The analytical process was reviewed using Baume and Clarke's 15-point checklist (Braun & Clarke, 2006).

### Language

Language and translation present particular challenges in qualitative research (Squires, 2009). For this reason we conducted, transcribed, and analysed the interviews in Norwegian. The quotations were only translated into English after selection for inclusion in the text (by the second author, a Norwegian fluent in English, in consultation with the first author, a native English speaker with rudimentary Norwegian). Given the importance of the quotations in the study, it was deemed desirable to check the translation accuracy (Santos, Black, & Sandelowski, 2015). The quotations were therefore retranslated back into Norwegian by an independent person (fluent in both languages, experienced in HAT, and familiar with the language of young substance users). No major differences in meaning were identified in the retranslation.

### Ethics

All necessary patient consent and data inspection authority approvals were obtained as part of the department's Youth Addiction Treatment Evaluation Project. The study was reviewed and approved by the Norwegian Regional Committee for Medical Research Ethics and performed according to their guidelines and the Helsinki Declaration. Patient participation in the research was voluntary, and all participants signed the required informed consent.

Patient details such as gender, age, and name are not used for reasons of anonymity and confidentiality. Instead, when attributing quotes, we simply refer to patients by number (P1 to P8). Similarly, the names of the horses, which in some cases could identify the patient, have been omitted.

## Results

Analysis of the data resulted in three main themes, namely *relationship with the horse*, *emotional effect*, and *mastery*. These three interrelated themes, together with nine sub-themes, reflect the participants’ experience of working with horses as an integral part of SUD treatment. Words such as “happy,” “calm,” and “safe” were used frequently in descriptions of different perceptions of the relationship with the horse and/or its role in the therapy process. Our thematic structure and use of quotations reflect the reality of the interrelated nature of the experiences described by the participants.

### Relationship with the horse

All except one of the participants (P6, who had participated in only one HAT session) emphasized that their relationship with one or more of the horses was one of the most important characteristics of HAT. The relationship was described as being “special and mutual,” “communicative,” and “like a friendship.”

#### Special and mutual

Most participants said that they had a favourite horse, or that one horse meant more to them than the others. Several participants described their first meeting with their favourite horse as “choosing one another,” often saying that they chose a particular horse (or the horse chose them) because of perceived similarities between them—“I saw a lot of myself in him, in a way. So I chose him” (P2). Others said they perceived a special sense of togetherness or mutuality—“I felt that he recognized me, and that we are good together, and we trust each other, both of us” (P5).

All but one participant described their interactions with the horses as involving a mutual, emotional relationship that was important to them and to their therapeutic process. Emphasis was placed on the horse appearing to understand and accept them without judgement—“You don't need to be popular to make the horse like you. The horse accepts you, like, for who you are” (P1). This perceived sense of mutuality in their work with the horses also involved mutual respect—“I feel that it is a partnership” (P2); and it was often seen as involving humour, which they also described as mutual—“It's just fun, when they push you with their nose, and mess with you. You can feel that the horse likes it” (P7).

#### Communicative

A sense of special communication was common throughout participants’ descriptions of their relationship with the horse. Several participants mentioned that they talked to the horse and felt that the horse understood them. This understanding was not necessarily expressed by the participants as the horse's semantic understanding of what they said but as a possible understanding of their emotional state, and sometimes in contrast to human understanding. “Like, if you let people know you've had a pretty bad day, I feel that's easier for the horse to handle, than for a person” (P3).

Many participants added an additional, specific dimension to their communication with the horse, namely, that it seemed to reflect or “mirror” their own behaviour and emotions.

The horse will let you know, or show, mirror my movements. It's just like you can see yourself a little bit, in the horse, and learn, get to know yourself by seeing what impact you have on the horse. (P8)

This description was often intertwined with reflection on a perceived meaning or impact.

It's just like the horse can understand what the human feels when the human acts. And based on that, you can think the other way around as well, that you can see it on the horse, when it expresses its own emotions. (P8)

#### Like a friendship

Most described the horse as being like a friend but mentioned that relating to the horses was different from relating to humans. This was described as part of the horses’ appeal. Physical contact, communication, and appearing to be unconditionally accepted by the horse were mentioned as illustrations of the differences between their relationships with humans and with horses. They described the horses as animals with personality, but not necessarily a human personality. However, they used terms and expressions about human relationships when they talked about the horses. “We are friends, really good friends. I don't know if I can use those words, but I feel [pause] attached to them. (…) Maybe (like) my girlfriend. The closest comparison I can think of” (P2).

### Emotional effect

All participants used words that indicate positive emotional effect when working with the horses. The key emotional effects linked to the horses included “feeling good,” “feeling tranquil,” and “feeling self-aware.” These were often described in relation to each other, with some participants emphasizing that they had to turn their focus away from themselves and onto the horse. The effect, they said, was similar, but in a positive way, to the effect of drugs. It enabled them to focus on something other than their personal problems.

In the beginning, it's happiness. Very positive. At the same time it kind of makes me forget everything. Actually, it's crazy [pause] because you can compare it to drugs. Because when you are high, you want to forget everything else and just be in your own little world. And not think about the negative stuff and your worries. The same thing happens when I'm here. Like, because then I'm only focused and only think about what happens right here, right now, and with the horse. (P2)

#### Feeling good

Expressions of happiness and fun occurred frequently and robustly through all the participants’ accounts of their interaction with the horses.

I just like to be with the horse. Because it is really fun. It is kind of playful. And you can think of other things. To be close to a horse, I think that's really, really good. I just become happy. And I feel calm, and, yes, it just feels good. (P8)

These accounts were characterized by certain vagueness, with the feeling described as of uncertain origin. While participants generally were unable or unwilling to say anything about why it made them happy to be with the horses, the majority expressed the need to communicate the pleasure of their experience with the horse(s). “It makes me happy, (…) just to groom the horses or take a walk with them or that kind of stuff” (P3). Some linked it to the interaction with the horses (a silent feeling of joy when the horse put his head upon the participant's shoulder). Others attributed it to specific activities, like grooming, feeding, or riding.

#### Feeling tranquil

Many participants also described being calmer with the horses, having a sense of tranquillity and security: “When I go out riding, no matter which horse I ride, then I have time to sort my thoughts, in a calm way that doesn't stress me out” (P1). Some sensed this with a particular horse (often their favourite), whereas others associated the feeling with just being with the horses in general: “And yes, just standing beside a horse. I don't know what it does, it just makes me [feel] really secure and happy, in a way” (P3). Most described this feeling as a positive emotion that was not necessarily in conflict with the thrill and excitement of being with a powerful and active animal. Thus, it is possible to interpret their statements about tranquillity and security as expressions of their relationship with the horses.

One participant described the calming effect of the horses as being similar to the effect of medication on anxiety and depression.

It's because of [horse's name] that I get rid of my anxiety. My anxiety and uneasiness and depression. That's, that's the worst thing in my life. I have been through pretty tough times because of it. So that kind of disappears and eases up a lot when I'm with [horse's name] or [horse's name]. (…) The horses mean a lot to me, because, in a way, they are the reason that I don't get anxious. I've been on tranquilizers, [name of medication] and different stuff when I was here last time. But to be with the horses makes you just as calm. In a better way. (P5)

Another participant described how he was able to meditate when riding and compared it to listening to music, which was something special to him.

What is interesting about this sub-theme, particularly for those with no previous experience of horses, is that participants described increased tranquillity when they were with a large, potentially dangerous animal. As indicated below, nervousness or fear, particularly initially, was mentioned by most participants but that was more in the context of achievement than as an emotional response.

#### Feeling self-aware

Participants also described better awareness of their own emotions and better body control as a result of their interaction with the horses. They described how the horses appeared to respond to their behaviour in a way that made their own behaviour easier to understand and regulate. They said that by learning about how their own behaviour appeared to affect the horse, they recognized that it was possible and even manageable to change their own behaviour.

If I'm calm then the horse is calm and if I make a sudden movement or think of something else, and appear to be unfocused, or just mess around, the horse will be like that as well. So horse therapy means a lot to me in a way because I have to be present and consistent. And then when I am, that's a pretty good state of mind to be in. (P8)

Participants described how when they learned more about the horses and their reactions to human behaviour and emotions, they also experienced how the horse could teach them about their own behaviour. For patient 3 it was emotional regulation: “I feel something sort of happened to me. I've become more positive and, yes, more sure of myself, of keeping calm and not making sudden movements.” For patient 4, it was caring: “Maybe I have learned to care more for other people and learned to take responsibility, both for myself and for others”. For patient 2 it was to be constructive: “Instead of getting mad and sitting on your hind legs, so to speak, you find the solutions.”

### Mastery

Most participants at some point used the word “mastery” (*mestring*) or a similar term to describe aspects of their work with the horses. It was the most frequently mentioned therapeutic value arising from the relationship with the horse. However, their meanings of the word varied. Some described a rewarding experience of managing an animal of such size, synonymous with “control.” Others emphasized succeeding in tasks through perceived cooperation with the horses, thus indicating a meaning of “achievement.” The participants that described feeling nervous or afraid when with the horses conveyed a third meaning, namely, coping with their own nervousness and “overcoming fear.” All three meanings capture a sense of self-efficacy, success, and empowerment—with the horse, with a task, with self.

#### Control

The participants who emphasized mastery of the horse described it in terms of a challenge to manage or control the horse.

When I lead her around, I try to walk in the front and so on. I feel that it is important that I be in charge. For her sake as well as mine. That I don't let her control me. It is about teaching me something. Learning to be firm and decisive. (P4)

#### Achievement

The sense of achievement was generally described in terms of personal attainment.

He was completely different from the other horses. So at first, when I started with him, I felt like, wow, really huge. But me and him, like I said I gained control over him pretty fast. And it was the first horse I managed to ride properly. Where I managed to sit properly when he galloped and everything. It felt right. (P2)

It was also usually acknowledged as occurring because of a perceived cooperative endeavour: “Horse therapy is a kind of partnership between human and horse. (…) The human's mastery with help from the horse” (P. 8). The rewarding emotional effect of achieving what seemed to be a collaborative partnership with the horse was also recognized.

I feel that they give you a pretty good feeling of mastery. When I am able to be with the horse by myself and feel that it is [pause] yes, safe and, and that I'm secure at the same time. It creates this kind of personal space, which feels very good. Where I can team up with an animal which is with you, in a way. (P8)

#### Overcoming fear

The emotions of nervousness, anxiety, and fear were frequently mentioned but usually described as feelings experienced when participants first met the horses or as a manageable challenge to be overcome. Participants cited the horses’ size, strength, and sudden movements as the reasons for their nervousness.

If they get startled and spook and stuff, then I'm afraid they will tread on me. That's really what I fear most, to be kicked or tread upon. But from what I've seen of the horses here, they are very kind. So it isn't the first thing that strikes me that they will walk over me. (P3)

Mastering fear of the horse, as a large and possibly frightening animal, was mentioned by most participants. It was also linked to the enjoyment and lure of the challenge of the large animal and/or doing something new.

## Discussion

To increase understanding of the HAT treatment process, including why patients in the HAT programme have been found to remain in SUD treatment for longer with a better chance of completing their treatment (Kern-Godal, et al., 2015), we examined in detail the participants’ own descriptions of how they experienced their relationship with the horses. The participants’ experiences and the meanings they attribute to them are part of the treatment process because they are experienced as characteristic of both the patient–horse relationship and the social reality of undergoing SUD treatment. Their genuine and detailed accounts contribute to an understanding of the role of the horse in the SUD treatment processes and its possible contribution to retention in treatment.

### The role of the horse

Participants described a relationship with one or more horses, which they valued as understanding, non-judgmental, emotional, and fun, but also as educational and therapeutic. These sentiments are generally prevalent in HAT theoretical literature (Bachi, 2013; Burgon, 2014; Hallberg, 2008; Lee et al., 2016; Lentini & Knox, 2015). They also occur in qualitative studies of horse ownership and recreational activities with horses (Davis, Maurstad, & Dean, 2015; Keaveney, 2008; Schuurman, 2014).

There are similarities between the basic concepts of attachment theory (Ainsworth, Blehar, Waters, & Wall, 1978; Bowlby, 1988; Wallin, 2007) and the central features of equine-assisted psychotherapy, such as security, affect mirroring, mentalization or reflective functioning, non-verbal communication, and body experience (Bachi, 2013). Specific examples of attachment-related processes involving the horse are evident in our study. For example, growing “attachment” is indicated in participants’ accounts of feeling “understood,” “secure,” and “calm” and their perceptions of the horse's affirmation of the individual. “Mentalization” (reflective functioning) can be seen in their understanding of how recognition of the horse's reactions to them could influence their own behaviour (mirroring). “Alliance” is evident in their accounts of trust, “feeling good,” growing empowerment, and achievement in partnership with the horse. These findings are not just consistent with the developing HAT theoretical literature (Bachi, 2013; Burgon, 2014; Hallberg, 2008; Lee et al., 2016; Lentini & Knox, 2015). They also resonate with certain theories of addiction treatment, most notably with those related to attachment (Flores, 2006) and therapeutic alliance (Miller & Moyers, 2014).

### Relevance of the patient–horse relationship to SUD

Addiction and attachment are reported to be frequently associated (Fletcher, Nutton, & Brend, 2015; Flores, 2006). Impaired attachment is a risk factor for SUD (Flores, 2006) in which alcohol and/or drugs can become a “solution.” The substances become in effect a “substitute attachment object” that may reduce the discomfort associated with impaired attachment, but they also become a cause of increased alienation from purposeful human interaction and relationships (Caspers, Cadoret, Langbehn, Yucuis, & Troutman, 2005).

Less effective psychotherapies have been characterized by lower levels of therapist empathy and less patient involvement (Roth & Fonagy, 2013). In SUD, impaired attachment can add additional challenges to productive patient–therapist alliance due to trust and abandonment issues (Shorey & Snyder, 2006) and can therefore necessitate extended treatment (Flores, 2006). In contrast, early therapeutic alliance “appears to be a consistent predictor of engagement and retention” (Meier et al., 2006, p. 304). Flores (2006) pointed to the benefits to be derived from addiction treatment approaches that increase affective bonds and repair associated disruptions arising from poor attachment.

Bachi (2013), drawing on Bowlby (1988) and Wallin (2007), illustrated HAT's relevance as an alliance-building mechanism that “highlights intimate bonds, the non-verbal realm, and the relation of the self to experience” and “can offer opportunities for restoring emotional capabilities and healing” (Bachi, 2013, p. 189). Consistent with her theory, our participants’ experiences appear to indicate that incorporation of the HAT intervention into SUD treatment enabled them, in a relatively short period, to experience a non-threatening, rewarding therapeutic relationship with the horse(s).

The horse–human relationship in HAT may be compared to that of an alliance between therapist and client, in which the client perceives in the horse an unconditional acceptance and empathy—a therapeutic relationship not unlike that described by Carl Rogers (1961) in which empathy and trust are fundamental. Consistent with Rogers’ theory, our participants’ early perceptions of the horse's unconditional acceptance and of their partnership or alliance with the horse(s) may have set the scene for broader rapport and therapeutic relationship. Other theoretical and qualitative studies of HAT indicate that the horse facilitates the relationship between the patient and the therapist (Burgon, 2014; Carlsson, 2014; Hallberg, 2008; Karol, 2007). It is interesting that none of our participants mentioned this, although our therapists contended that it was true of the horses they work with. More scientific studies of the interactions within the horse–client–therapist triad and examination of whether the horse is in effect a mediator of improved relationship and alliance with the human therapist certainly warrant further investigation.

Miller (2000) compared motivational interviewing to the collaborative working relationship found in horse training. In each, he identified relaxed “collaborative moving-with” partnerships, which are “directive, yet highly supportive and attuned to the moment to moment concerns of the other” (Miller, 2000, p. 288). Our participants described processes and relationships with the horses and attributed meanings to that relationship that are congruent with Miller's assertions.

The object of this study was to explore how participants made sense of their relationship with the horse and its role in and relevance to their treatment. These SUD patients described their relationship with the horses and its therapeutic role as a genuine and positive reality. They indicated that the horse is experienced as being possibly more understanding than humans. As such the horses enabled them to explore issues and to recognize behavioural challenges in a way they described as safe, insightful, and pleasurable. Security and insight are important components of healthy attachments. Like pleasure, they are recurring themes in studies of recreational activity with horses (Davis & Maurstad, 2016). Pleasure and positive emotion are not unquestioned in psychotherapy (Stalikas & Fitzpatrick, 2008). However, they can, as Fitzpatrick and Stalikas (2008) concluded from their analysis of Fredrickson's (2001) work on emotion in positive psychology, be both indicators of and “generators of change” (Fitzpatrick & Stalikas, 2008, p. 137).

If the horse lends a measure of pleasure and contributes to endurance in working with difficult themes, thereby contributing to therapeutic alliance, behaviour change, and retention in treatment, its contribution to SUD is not insignificant. Its potential for contributing to positive post-treatment, on-going recovery, and relapse prevention should be further explored.

The horses appeared to generate a sense of positive attachment and alliance relevant to SUD treatment. This unique study, in a growing but uncharted area of SUD treatment, therefore has important and relevant clinical implications. Its strengths are its naturalistic design; the strong clinical, research, and managerial backgrounds of its researchers; and its consistency with both HAT (Bachi, 2013) and addiction (Flores, 2006) theory.

### Methodological considerations

We deliberately used extensive descriptive quotations from participants because they can contribute new knowledge and understanding to an innovative therapy that is still relatively unknown in the health sector. Furthermore, when reported, it is usually from the provider's perspective. Patients can provide new information (Laudet, 2007; Neale, 1998; Skatvedt & Schou, 2010) relevant to examination of the SUD treatment processes as advocated by Miller and Moyers (2014), Flores (2006) and Roth and Fonagy (2013).

The findings were derived from a study that used translated data from a small number of participants to explore the specific experience of a specific activity within a specific context. As many SUD patients drop out early in the treatment process it was important to include at least one participant with little experience of HAT in the study. That participant, P6, who had no previous experience of horses, spoke about other aspects of HAT but said little about the horses, perhaps indicating insufficient time for relationship building. In addition, some patients were not included in the study because their therapist believed the interview could be detrimental at that point in their recovery process, so our findings may not be applicable to new or “fragile” patients.

Measurements of attachment and therapeutic alliance would have added internal validity, but regrettably none were available for the participants. Similarly, as Patton (2015) advocated, comparison with negative or different findings can add validity in qualitative studies. Despite searching, we found none, in either our own or other HAT studies, with which to make comparisons. This positive/negative imbalance in HAT research is a challenge in need of further investigation.

Language is another possible shortcoming (Squires, 2009; Temple & Young, 2004). All data collection and analysis were carried out in Norwegian, presenting challenges to the first author's rudimentary Norwegian. Working closely with the second and third authors, she was aware of a certain dependence on their interpretations of nuanced meanings throughout the study process.

Interpretation is another challenge common to most qualitative studies. Patients’ insights and accounts of those insights can be influenced by many factors, including the social context of the experience and researchers’ interpretations of the insights (Malterud, 2001; Patton, 2015). Although the fourth and fifth authors provided a certain counterbalance, the first three authors’ pro-horse backgrounds undoubtedly influenced the study choice, process, interpretations, and outcomes. Throughout, they discussed their shared and different views and were cognizant of the need to discuss and bracket preconceptions.

The above methodological considerations indicate a need for caution regarding transferability to other treatment domains. Further research is needed into variance within and between substance use and other specific diagnostic groups to determine those most responsive to HAT (Lee et al., 2016). In addition, the nature of the therapeutic and change processes in HAT needs further exploration and testing to strengthen the theoretical basis (Bachi, 2012; Dell et al., 2011).

## Conclusion and practice implications

In this phenomenological study, thematic analysis was used to investigate how the participants constructed the reality of their relationship with the horse(s) and their perception of the consequences of that reality in SUD treatment. The findings were interpreted within an attachment theory context.

We concluded that the patient–horse relationship in HAT may facilitate positive attachment, reflective functioning, and emotional regulation. As indicated in other studies, the positive relationship experienced with the horse may also facilitate strengthened alliance with the HAT therapist. The patients’ rich descriptions indicated that their relationship with the horses and the therapeutic implications of that relationship were important factors in their treatment process. The horses appeared to be facilitators of a positive self-construct and were an important emotional support during treatment. The participants’ experiences add to our understanding of the impact of HAT on SUD treatment and, while not conclusive, the very positive nature of the experience may be relevant to SUD treatment retention and completion. As questions remain about the neuro-biological nature of the human–horse relationship and about the actual therapeutic processes involved, relevance to other treatment domains cannot be assumed. However, the application of HAT as an adjunct therapy for SUD and other psychological illness, including its possible contribution to positive post-treatment, on-going recovery, and relapse prevention in community-based settings, warrants further study.
